# Highly-Responsive Broadband Photodetector Based on Graphene-PTAA-SnS_2_ Hybrid

**DOI:** 10.3390/nano12030475

**Published:** 2022-01-29

**Authors:** Guigang Zhou, Huancheng Zhao, Xiangyang Li, Zhenhua Sun, Honglei Wu, Ling Li, Hua An, Shuangchen Ruan, Zhengchun Peng

**Affiliations:** 1 Center for Stretchable Electronics and NanoSensors, College of Physics and Optoelectronic Engineering, Shenzhen University, Shenzhen 518060, China; 2176285304@email.szu.edu.cn (G.Z.); zcpeng@szu.edu.cn (Z.P.); 2 Key Laboratory of Optoelectronic Devices and Systems of Ministry of Education and Guangdong Province, College of Physics and Optoelectronic Engineering, Shenzhen University, Shenzhen 518060, China; szh@szu.edu.cn (Z.S.); hlwu@szu.edu.cn (H.W.); 3 Shenzhen Key Laboratory of Laser Engineering, College of Physics and Optoelectronic Engineering, Shenzhen University, Shenzhen 518060, China; 1800281011@email.szu.edu.cn (H.Z.); scruan@szu.edu.cn (S.R.); 4 Key Laboratory of Advanced Optical Precision Manufacturing Technology of Guangdong Higher Education Institutes, College of Applied Technology, Shenzhen University, Shenzhen 518060, China; 2170285209@email.szu.edu.cn

**Keywords:** flexible photodetector, SnS_2_ nanosheets, high responsivity

## Abstract

The development of wearable systems stimulate the exploration of flexible broadband photodetectors with high responsivity and stability. In this paper, we propose a facile liquid-exfoliating method to prepare SnS_2_ nanosheets with high-quality crystalline structure and optoelectronic properties. A flexible photodetector is fabricated using the SnS_2_ nanosheets with graphene-poly[bis(4-phenyl) (2,4,6-trimethylphenyl) amine (PTAA) hybrid structure. The liquid-exfoliated SnS_2_ nanosheets enable the photodetection from ultraviolet to near infrared with high responsivity and detectivity. The flexible broadband photodetector demonstrates a maximum responsivity of 1 × 10^5^ A/W, 3.9 × 10^4^ A/W, 8.6 × 10^2^ A/W and 18.4 A/W under 360 nm, 405 nm, 532 nm, and 785 nm illuminations, with specific detectivity up to ~10^12^ Jones, ~10^11^ Jones, ~10^9^ Jones, and ~10^8^ Jones, respectively. Furthermore, the flexible photodetector exhibits nearly invariable performance over 3000 bending cycles, rendering great potentials for wearable applications.

## 1. Introduction

Flexible optoelectronic devices have attracted considerable attentions due to their potential applications in wearable systems [1,2,3,4], imaging sensing [5], and communications [6], where especially flexible broadband photodetectors with high responsivity and stability are highly desired. However, the complexity and high cost of traditional rigid materials limit their extensive applications in flexible devices [7]. In the past decades, two-dimensional (2D) materials, such as phosphorenes, transition-metal dichalcogenides (TMDCs), and IV-VI group semiconductors, have been widely investigated in solar cells [8], photodetectors [9,10,11], etc. Moreover, 2D semiconductors are particularly suitable as active channel materials in wearable optoelectronic devices owing to their atomically thin structure, mechanical flexibility, strong in-plane covalent bonding, and excellent electrical and optoelectronic properties [12,13]. In addition, their compatibility with other materials, including organic semiconductors [14,15], quantum dots [16,17], nanosheets [18], perovskites [19], etc., is conducive to form heterojunctions with splendid properties. These hybrid heterostructures can significantly improve the device performance compared with that of individual materials. Zhou et al. have demonstrated a broadband photodetector based on self-encapsulated graphene-black phosphorus (BP) nanosheets and obtained a high responsivity of 7.7 × 10^3^ A/W [20], while the photodetector based on few-layers BP exhibited a relatively low responsivity of 4.8 mA/W [21]. Li et al. realized monolayer graphene-SnSe_2_ QDs-based ultraviolet-detector with a responsivity of ~7.5 × 10^3^ A/W [22].

As a typical 2D TMDC, tin disulfide (SnS_2_) has a wide bandgap within the range of 2.08–2.44 eV [23,24] and displays a competent experimental mobility of 230 cm^2^ V^−1^ s^−1^ [25]. It is promising for sustainable optoelectronics and catalysis applications because of the extraordinary electronic and optical properties, earth-abundance, and environment-friendliness [26,27,28,29,30,31]. SnS_2_ nanosheets have been intensively investigated in photodetectors because the efficient light absorption properties can generate an adequate number of carries under illumination compared to the corresponding bulk counterparts. Su et al. exhibited a photodetector based on SnS_2_ thin crystal arrays by chemical vapor deposition (CVD), and the device showed a responsivity of 8.8 mA/W at 457 nm [27]. In practice, most of reported SnS_2_-based photodetectors suffer from a low responsivity and a narrow photoresponsive range which, hence, limits their practical applications [32]. Zhou et al. successfully synthesized single-crystal SnS_2_ nanosheets via CVD using a low-melting-point precursor and fabricated flexible phototransistors with a high responsivity up to 34.6 A/W [33]. Besides improving the crystalline structure, oxygen-plasma treatment was proposed to improve the carrier activity by introducing more traps to the mechanical-exfoliated SnS_2_ nanosheets [34]. Enhanced performance has been achieved with a high responsivity from 385 to 860 A/W in a broadband range. Constructing heterostructures is proved to effectively optimize the responsivity of SnS_2_-based photodetectors [11,35]. Li et al. stacked the hexagonal SnS_2_ with orthorhombic SnS flake through a one-step CVD method for a vertical SnS_2_/SnS heterostructure, and the obtained photodetectors demonstrated a high optoelectronic performance with a responsivity of 27.7 A/W [36]. Zhao et al. mechanically exfoliated the graphene and SnS_2_ to form a graphene/SnS_2_ van der Waals heterostructure in photodetectors, and they achieved a broadband photoresponse with a highest responsivity up to 7.7 × 10^3^ A/W at 365 nm [37]. However, the complexity, high cost and limited controllability for the fabrication of both 2D material layers in heterostructures with either CVD or mechanical-exfoliation hinder the applications of SnS_2_ in broadband photodetectors. On the other hand, a facile ethanol thermal method was applied to synthesize SnS_2_ nanosheet microspheres for flexible photodetectors [38]. Nevertheless, their photoresponsive performance still needs a long way for practical applications. High-performance photodetectors based on SnS_2_ nanosheets by a facile low-cost and large-scale fabrication are still rarely reported at present. It is highly desired to explore liquid-phase synthesis of SnS_2_ for photodetector applications not only for simplifying their fabrication to push one step torwards industrial applications but also offering a fundamental database platform for mechanism exploration and optimization of liquid-phase 2D-material-based optoelectronics.

In this paper, we have successfully synthesized high-quality SnS_2_ nanosheets in a mixed solution of water and ethanol via the liquid-phase exfoliation method [39,40]. The outstanding optoelectronic properties of the SnS_2_ nanosheets are applicable in flexible photodetectors. The photodetector based on the graphene-poly[bis(4-phenyl) (2,4,6-trimethylphenyl) amine (PTAA)-SnS_2_ hybrid with 100 um channel length is designed and shows an excellent performance with good flexibility and a broadband response from ultraviolet to near infrared wavelength. The maximum responsivity of the photodetector is 1 × 10^5^ A/W, 3.9 × 10^4^ A/W, 8.6 × 10^2^ A/W, and 18.4 A/W, and the specific detectivity can reach ~10^12^ Jones, ~10^11^ Jones, ~10^9^ Jones, and ~10^8^ Jones under 360 nm, 405 nm, 532 nm, and 785 nm illuminations, respectively. This hybrid photodetector shows a high responsivity and detectivity at low light intensity, coupled with a broadband photoresponse from 360 nm to 785 nm, and the highest responsivity is higher than the currently reported SnS_2_ nanosheet devices, especially for the solution-processed flexible SnS_2_ photodetectors. The excellent performance of the flexible devices remains relatively constant after bending over 3000 times, rendering a high bending endurance. These results indicate that the flexible photodetectors based on the hybrid structure can be featured as excellent candidates for flexible and wearable optoelectronic devices.

## 2. Materials and Methods

### 2.1. Synthesis of SnS_2_ Nanosheets

**Figure 1:**
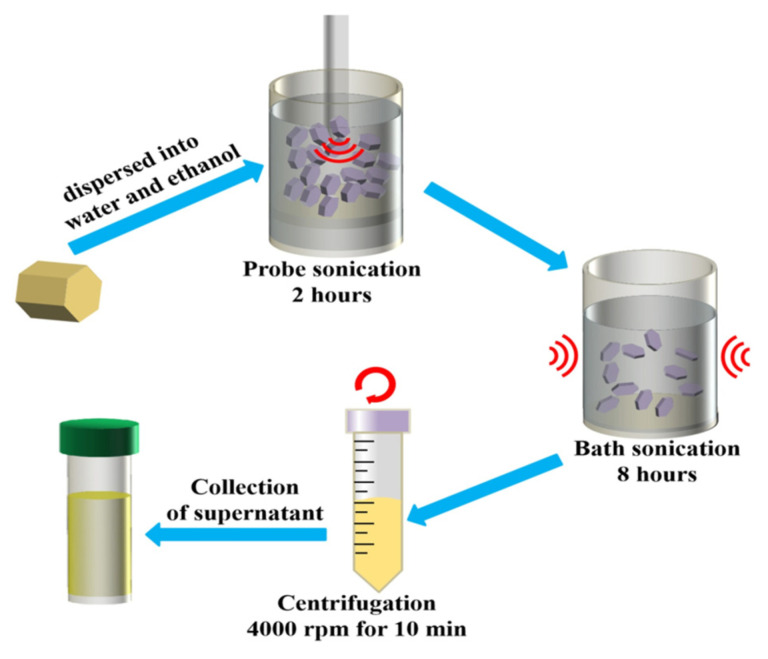
Schematic diagram of the fabrication process for SnS_2_ nanosheets.

SnS_2_ crystals were purchased from 6 Carbon Technology (Shenzhen, China). The fabrication process of SnS_2_ nanosheet solution is displayed in Figure 1. As for liquid exfoliation process, 50 mg SnS_2_ crystals were added into 50 mL mixed solution of water and ethanol (volume ratio = 7:3) inside a 60 mL glass vial. The mixed solution underwent an ultrasonication treatment at the power of 600 W with an ice-water bath to keep the temperature lower than 20 °C. The ultrasonication was conducted for 2 h with a pulse of on for 6 s and off for 4 s to protect the probe. Subsequently, the obtained solution was sonicated in water bath for 8 h at a frequency of 50 kHz and a power of 30 W. At last, the stock solution was centrifuged at 4000 rpm for 10 min, and then the top three-fourths of the supernatant were collected for further investigation.

### 2.2. Device Fabrication

Polyethylene terephthalate (PET) substrate was rinsed with acetone, ethanol, and deionized water for 5 min, respectively. Single-layer graphene film, purchased from 6 Carbon Technology (Shenzhen, China), was transferred onto the plasma-treated substrate via a wet transfer method [39]. Au (100 nm) electrodes were deposited by thermal evaporation with a shadow mask (W/L = 2 mm/0.1 mm), and the PTAA solution (3 mg/mL in chlorobenzene) was spin-coated on the top of graphene at a speed of 3500 rpm for 30 s and then heated at 100 °C for 20 min. The PTAA layer was treated with optimized plasma irradiation (argon and oxygen hybrid gas for 30 s) to increase the surface hydrophilicity. After that, the SnS_2_ nanosheet solution was spin-coated onto the PTAA layer at 1000 rpm for 10 s. Meanwhile, the PET-graphene-PTAA device regarded as the control sample was fabricated through a similar method as described above.

### 2.3. Characterization

The SnS_2_ nanosheet solution was drop-casted on Cu grid, and transmission electron microscopy (TEM) images were obtained by an FEI Titan Cubed Themis G2 300 instrument (FEI, Eindhoven, The Netherlands) equipped with an X-ray energy dispersive spectrometer (EDS). X-ray photoelectron spectroscopy (XPS) data of SnS_2_ nanosheets were characterized by a K-Alpha system (Thermo Fisher Scientific, Waltham, MA, USA). Raman experiments were conducted by Horiba Raman microscope (Labram HR Evolution, Horiba, Japan) with an excitation wavelength of 514.5 nm. X-ray diffraction (XRD) patterns were obtained by a Bruker D8 ADVANCE diffractometer (Bruker, Karlsruhe, Germany) with an X-ray generator (Cu target). The atomic force microscopy (AFM) images were collected by a Bruker Dimension Icon (Bruker, Karlsruhe, Germany). The absorption spectra for the samples of solution and on substrates were recorded by spectrofluorometer (FS5, Edinburgh, UK). Electrical characteristics of the devices were carried out with a Keithley 4200 semiconductor analyzer in a glovebox filled with nitrogen. The photoelectric properties of devices were measured under illumination for 30 s (illumination period: 30 s from the onset) by CHI successive lasers (360, 405, 532, and 785 nm) with a diameter of 9.5 mm and a distance of 8.4 cm. The mechanical stress was characterized with Fatigue Stretcher (Instron E1000, Boston, MA, USA).

## 3. Results

**Figure 2:**
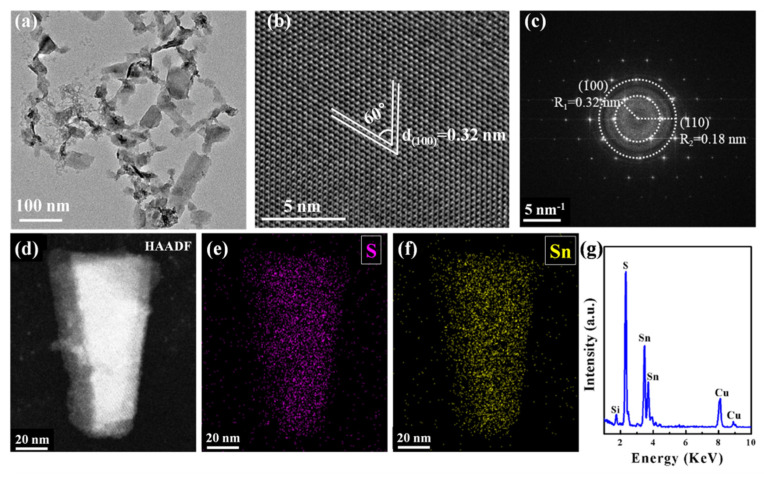
Morphology characterization of SnS_2_ nanosheets. (**a**) TEM image; (**b**) HRTEM image; (**c**) Selected area electron diffraction (SAED) pattern image; (**d**–**f**) TEM-EDS mapping of SnS_2_, Sn, and S in the nanosheets; (**g**) EDS energy dispersive spectrum of SnS_2_ nanosheets.

The TEM image in Figure 2a reveals the distributed lateral sizes and wrinkles of SnS_2_ nanosheets because of their atomically thin layer and two-dimensional planar structure. In addition, the high-resolution TEM (HRTEM) image in Figure 2b further exhibits a lattice spacing of 0.32 nm with a crystal plane angle of 60° in the nanosheet structure. This lattice distance is well identified as the (100) plane of SnS_2_, corresponding to the reported value of 0.317 nm of SnS_2_ crystal [27]. The selected area electron diffraction (SAED) pattern (Figure 2c) demonstrates a hexagonal structure of the single-crystal SnS_2_ nanosheet. Moreover, the lattice spacings of R_1_ and R_2_ rings are calculated to be 0.32 nm and 0.18 nm, which agree well with the (100) and (110) characteristic planes in SnS_2_ crystal [26]. The EDS mapping characterizes the elemental distribution of the as-prepared SnS_2_ nanosheets shown in Figure 2d–f. These images indicate that S and Sn elements are uniformly distributed in the nanosheet, and the EDS spectrum in Figure 2g shows clear peaks of S and Sn elements with an atomic ratio of ≈ 2.35:1, where the Cu and Si elements are from the TEM grid [33].

**Figure 3:**
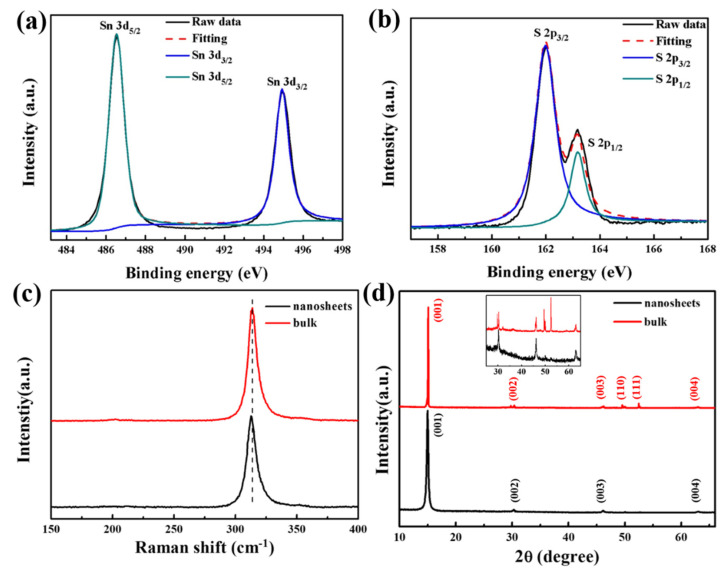
Spectral characterization of SnS_2_ nanosheets. (**a**,**b**) XPS spectra of SnS_2_ nanosheets. (**c**) Raman spectra of SnS_2_ nanosheets (black line) and bulk SnS_2_ (red line) excited by a laser of 514 nm. (**d**) XRD pattern of SnS_2_ nanosheets (black line) and bulk SnS_2_ (red line), and inset is the magnified XRD spectra in the large angle region.

The XPS spectra of the SnS_2_ nanosheets are demonstrated in Figure 3a,b, and the two binding energy peaks of Sn 3d at 486.5 and 494.9 eV correspond to the Sn 3d_5/2_ and Sn 3d_3/2_ (Figure 3a), respectively. An energy discrepancy of around 8.4 eV is observed between the two Sn 3d peaks that is characteristic for tetravalent Sn 3d states. Additionally, the peaks at 161.9 and 163.1 eV corresponding to S 2p_3/2_ and S 2p_1/2_ are illustrated in Figure 3b, which is in agreement with S at a state of −2. The fitting peaks separated by a typical value of 1.2 eV match well with previous reported values [26]. These results indicate that the SnS_2_ nanosheets possess a good purity and a high crystal quality. Raman spectroscopy is an authoritative and nondestructive method to characterize the structure and vibrational modes of 2D materials. The Raman spectra of SnS_2_ in bulk and nanosheets are exhibited in Figure 3c, where the strong characteristic peak at 313.4 cm^−1^ is recognized as A_1g_ mode [27]. Compared to the bulk SnS_2_, the A_1g_ peak of the SnS_2_ nanosheets displays a redshift of around 1 cm^−1^ relating to the significant reduction of scattering centers for the in-plane scattering, and the peak intensity declines as the thickness decreases. The crystal structure is further characterized by XRD in Figure 3d, and the primary diffraction peaks of bulk SnS_2_ at 15.02°, 30.35°, 46.12°, 50.00°, 52.49°, and 62.99° are well indexed to the (001), (002), (003), (110), (111), and (004) planes of a hexagonal SnS_2_ (space group = *p*$\bar{3}$*m*1, PDF no. 23-0677). Compared with that of bulk SnS_2_, the characteristic (001) peak of SnS_2_ nanosheets becomes broader, and some other peaks disappear (shown in the inset of Figure 3d), because of the lattice expansion and nanocrystalline structure [20].

**Figure 4:**
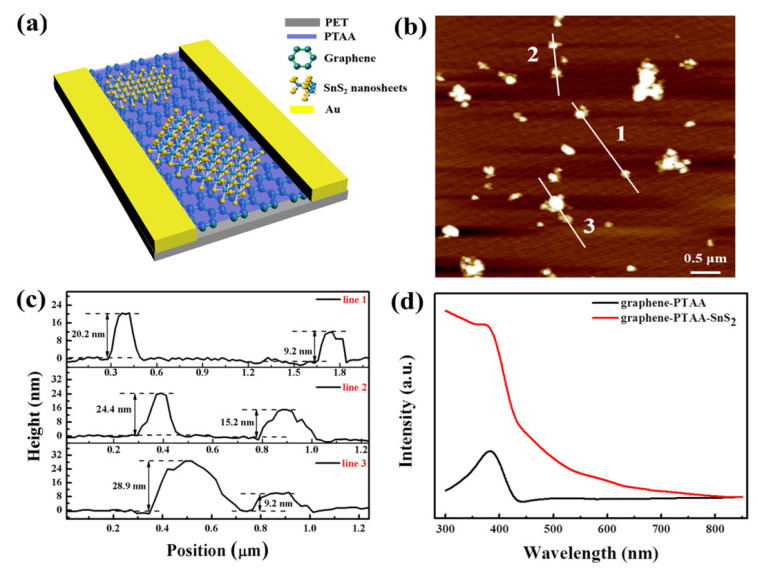
Characterization of graphene-PTAA-SnS_2_ nanosheet hybrids. (**a**) Schematic diagram of the photodetector based on graphene-PTAA hybrid decorated with SnS_2_ nanosheets. (**b**) AFM image of the SnS_2_ nanosheets with three marked lines. (**c**) Thickness profiles corresponding to three lines in (**b**). (**d**) Absorption spectra of graphene-PTAA and graphene-PTAA-SnS_2_ nanosheets.

The schematic of the flexible graphene-PTAA-SnS_2_ hybrid photodetector is illustrated in Figure 4a. The AFM image (Figure 4b) reveals the representative morphology of the comparatively uniformly dispersed SnS_2_ nanosheets in the 100 µm channel, and the height profiles from the three selected lines are applied to measure the thickness quantitatively. As shown in Figure 4c, the corresponding thickness from the lines (Figure 4b) is ranging from 9 to 30 nm, indicating that the 2D SnS_2_ nanosheet flakes embrace various sizes and thicknesses from the low centrifugation speed. In addition, the morphology of SnS_2_ nanosheets in the channel is also displayed in the SEM images (Figure S1a). It is worth noting that the graphene-PTAA hybrid layers are not entirely covered by SnS_2_ nanosheets, and the stacking of nanosheets is observed at some places in the channel. This may lead to a nonuniform photocurrent distribution in the channel, however, the general performance of the hybrid photodetector shows little obvious spatial dependence for different device units and batch-to-batch fluctuation since the SnS_2_ nanosheets can be regarded as relatively uniformly distributed on the PTAA film compared to the long length of the channel (100 µm). The absorption spectra of the graphene-PTAA-SnS_2_ nanosheets and the graphene-PTAA are illustrated in Figure 4d. Note that obvious light absorption increases in the short-wavelength range by comparing the absorption intensity of the graphene-PTAA-SnS_2_ nanosheets with that of the control sample (graphene-PTAA), while very weak absorption is observed in the long-wavelength range. The enhanced absorbance of the graphene-PTAA-SnS_2_ hybrid in the UV-visible range is mainly ascribed to the strong absorption of the SnS_2_ nanosheets as compared with the spectrum of bare SnS_2_ nanosheet solution in Figure S1b. The absorption edge of the SnS_2_ nanosheet solution is about 590 nm (Figure S1b). The relationship of (αhν)^1/2^ VS hν is displayed in the inset of Figure S1b, wherein h, ν, and α values represent the Planck constant, photon frequency, and optical absorption coefficient, respectively. The energy band gap (Eg) is the intercept to extrapolate the fitting line on the horizontal ordinate in the absorption spectrum, and the obtained band gap of ~2.1 eV is consistent with the absorption edge of 590 nm [41].

**Figure 5:**
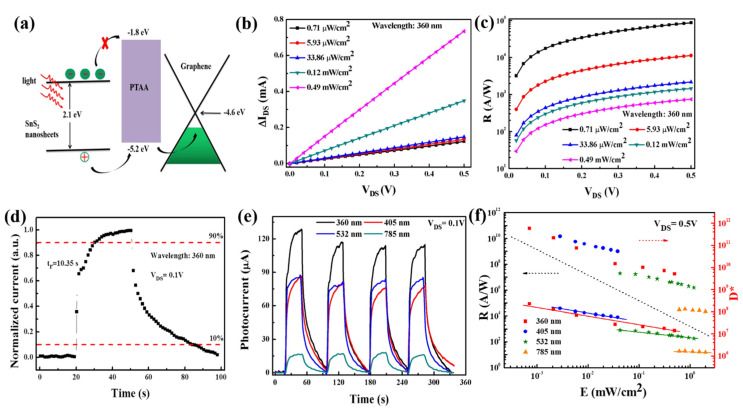
Performance of the graphene-PTAA-SnS_2_ hybrid photodetector. (**a**) Charge transfer diagram in graphene-PTAA-SnS_2_ hybrid device. (**b**) Photocurrent and (**c**) responsivity as a function of V_DS_ (0–0.5 V) under different irradiance at 360 nm. (**d**) Normalized time-dependent photocurrent of the device at 360 nm laser with the maximum intensity (0.49 mW/cm^2^). (**e**) Time-dependent photoresponse under on/off illumination at different wavelengths of 360, 405, 532, 785 nm (voltage: 0.1 V) with the corresponding highest light intensity (0.49, 0.038, 1.19, and 2.01 mW/cm^2^). (**f**) Responsivity and specific detectivity as a function of light intensities with varied light sources (360, 405, 532, and 785 nm).

To elucidate the structure design and working mechanism of the device, carrier transfer diagram under illumination is demonstrated in Figure 5a. In this structure, graphene works as the carrier transfer layer, while the SnS_2_ nanosheets function as the light harvesting materials. A p-type organic semiconducting PTAA layer with a bandgap of 3.4 eV is introduced, and it can not only act as a medium to improve the hydrophilicity of the device for spin-coating of SnS_2_ nanosheets without damaging graphene, but more importantly, enhance the separation of photo-generated carriers as a hole transport layer [42,43]. Photo-induced holes in SnS_2_ nanosheets can be transferred to graphene via the PTAA layer on account of the lower energy level. However, the transfer of electrons is suppressed owing to the higher unoccupied molecular orbital (LUMO) energy level of PTAA. In addition to the favorable energy band alignment of the heterojunction, abundant trap states from the stacking boundaries and defects of liquid-exfoliated SnS_2_ nanosheets can capture electrons easily, resulting in a strong photogating effect in the channel of graphene-PTAA-SnS_2_ heterostructure [44]. As shown in Figure 5b, the linear relationship between photocurrent and V_DS_ shows an excellent ohmic contact under varied radiation intensities at 360 nm, and the value ∆I (∆I = I_light_ − I_dark_, where I_light_ and I_dark_ are the drain currents under illumination and dark conditions) gradually increases with increasing the radiant intensity. Meanwhile, the ∆I as a function of V_DS_ under 405 nm, 532 nm, and 785 nm are displayed in Figure S2a–c. Responsivity (R), the ratio of photocurrent to incident light power, is one of the indispensable figure-of-merits to evaluate the photodetector characteristics and can be calculated from the photocurrent and the incident light by the following formula:

(1) $$R\;\left(A/W\right)=\frac{\Delta I}{P}=\frac{\Delta I}{{\mathrm{WLE}}_{e}}$$

where ∆I is the photocurrent in the channel, E_e_ is the power density of the incident light, W and L are the width and length of the active area, as illustrated in Figure S3. The obtained responsivity at 360 nm gradually increases with the increasing of V_DS_ and the decreasing of light intensity in Figure 5c. Furthermore, the responsivity of the device with 405 nm, 532 nm, and 785 nm illumination are shown in Figure S2d–f. The maximum responsivity can be up to ~10^5^ A/W due to the excellent properties of the PTAA-graphene-SnS_2_ hybrid.

One-cycle normalized photocurrent response is displayed in Figure 5d. The sharp raise of photocurrent at the initial stage is attributed to the quick generation and separation of photo-induced carriers in the hybrid structure. Subsequently, the increase of current slows down until, finally, the photocurrent reaches saturation. A response speed of 10.35 s is observed, corresponding to the current rising from 10% to 90%. The relatively long rise and decay time for this hybrid photodetector is associated with the trapped charge carriers and prolonged excess carrier lifetime, which is the characteristic of photogating effect [45,46]. The widely existing trap states in the defects or interfaces of the heterostructure can capture the photogenerated electrons resulting in a negative gating to modulate the channel conductance. The process of releasing of the trapped carriers is very slow, and this will give rise to a long response time in the photogating effect [45]. In Figure 5e, the flexible device shows good reproducibility and repeatability of ON-OFF switching behaviors under different wavelengths, demonstrating a broadband photoresponse to various wavelengths. It is noted that the photocurrent at short wavelength is higher than that of long wavelength, consistent with their absorption property.

The specific detectivity (D*) is also a central parameter to estimate the performance of photodetectors, and it is given by [47,48,49],

(2) $$D^{*}=\frac{\sqrt{A\Delta f}}{\mathrm{NEP}}$$

where A is the effective area of the device in cm^2^, Δ*f* is the electrical bandwidth in Hz, and NEP is the noise equivalent power, which is defined as the minimum impinging light power that a photodetector can discern from the noise. Three sources of noise current, consisting of 1/*f* noise (I*_f_*, Figure S4), shot noise (I_s_), and thermal noise (I_t_), mainly contribute to the total noise current, and the NEP value can be calculated from NEP = $\frac{\sqrt{S_{I}}}{R}$ (the noise spectral density S_I_ = S_I*f*_ + S_Is_ + S_It_, where the detailed noise spectral density (S_I*f*_, S_Is_, S_It_) is estimated in the supporting information, and R is the responsivity) [50,51,52]. At room temperature and a modulation frequency of 1 Hz, NEP value of the hybrid photodetector is as low as 10^−11^~10^−15^ W Hz^−1/2^. The responsivity and specific detectivity (D*) as a function of varied radiant intensities at 360, 405, 532, and 785 nm are shown in Figure 5f. It is noted that both of the R and D* values decrease nonlinearly with the increase of the incident light intensity, implying the photogating effect. The dependence of R on the intensity of light (P) follows a relationship of R~P^β−1^ as rendered from the fitted lines in the logarithmic coordinates at various wavelengths. The scope of 0 < β < 1 is usually observed in low dimensional photodetectors [53], and the β is fitted to be 0.21 for 360 nm laser. The performance of photodetector in short wavelength range is better than that of the long wavelength owing to their better absorption. The device with 100 μm channel length shows the largest responsivity and the highest detectivity of up to 10^5^ A/W and ~10^12^ Jones under 360 nm laser with the lowest light intensity (V_DS_ = 0.5 V). Meanwhile, the poor performance of the control sample (graphene-PTAA film without SnS_2_ nanosheets) in Figure S5 also indicates that SnS_2_ nanosheets play an important role in the high responsivity of the photodetector. As a result, the excellent performance of the hybrid device is contributed to the combination of outstanding absorption of SnS_2_ nanosheets and efficient carrier transport of PTAA and graphene. The performance of the hybrid photodetector is compared with other reported photodetectors based on various 2D materials shown in Table 1, where we define the liquid-exfoliated method because of the solution-process for the photoactive SnS_2_ nanosheet fabrication. The high responsivity and detectivity of this hybrid device demonstrate that the liquid-exfoliated SnS_2_ nanosheets are promising candidates for photodetection applications.

**Figure 6:**
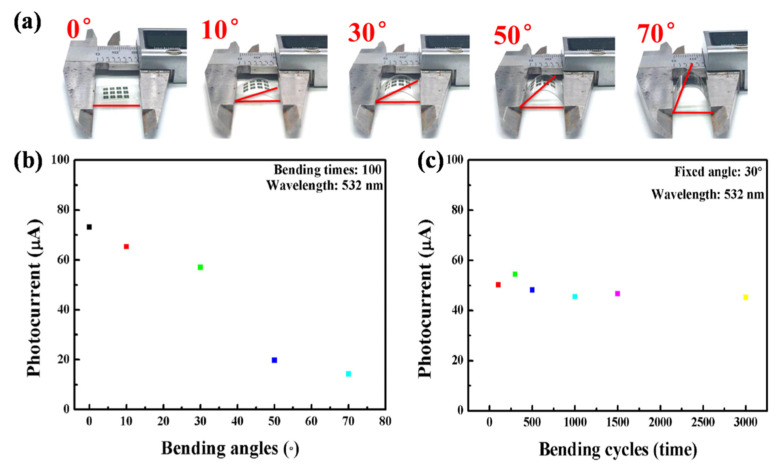
Performance of the flexible photodetectors. (**a**) Photographs of the device at different bending angles. (**b**) Evolution of photocurrent at various bending angles after 100 bending times (voltage: 0.1 V; light: 532 nm–1.19 mW/cm^2^). (**c**) Variation of photocurrent after different bending times at a fixed bending angle (voltage: 0.1 V; light: 532 nm–1.19 mW/cm^2^).

In order to apply the photodetectors to the flexible and wearable systems, bending endurance of the flexible device must be considered. The bending test of the device was conducted via a Vernier caliper as shown in Figure 6a [59]. Photocurrent after bending 100 times at different angles (10° to 70°) was measured at V_DS_ of 0.1 V under 532 nm, shown in Figure 6b. Higher bending angles lead to a more severe current degradation with a nearly linear trend. The relationship between the mechanical stress level of the films and the bending angles in Figure S6 demonstrates that the stress gradually increases along with the increase of bending angles. The results imply that the stress concentration plays an important role in the degradation of the device performance. In addition, the cyclic durability at a fixed bending angle of 30° is displayed in Figure 6c. Note that the photocurrent value remains approximately invariant even after 3000 cycles, indicating a good cycling stability and flexibility of the photodetectors.

## 4. Conclusions

In summary, SnS_2_ nanosheets have been prepared via a facial low-cost liquid-phase exfoliation method. The liquid-exfoliated SnS_2_ nanosheets reveal a high-quality crystalline structure and intriguing photoelectronic properties. The outstanding absorbance of SnS_2_ nanosheets is integrated with graphene-PTAA hybrids to realize a flexible broadband photodetector. The graphene-PTAA-SnS_2_ hybrid photodetector exhibits a high responsivity of ~1 × 10^5^ A/W and a specific detectivity of up to ~10^12^ Jones at a light intensity of 0.71 µW/cm^2^ with 360 nm laser. The flexible photodetector can endure 3000 bending cycles at a bending angle of 30° without obvious performance degradation. Benefiting from the low cost and eco-friendly fabrication, the liquid-exfoliated 2D SnS_2_ nanosheets have potentially extensive applications for optoelectronic devices.

## Figures and Tables

**Table 1:** Performance comparison of the graphene-PTAA-SnS_2_ hybrid photodetectors with previous reports based on 2D materials.

Material	Flexible (yes/no)	Fabrication	R (A/W)	D* (Jones)	References
SnS_2_ NSs-CVD graphene	yes	liquid-phase exfoliation	~10^5^	~10^12^	This work
Vertical SnS_2_ nanosheets	no	CVD	1.85	4.91 × 10^9^	[54]
single-crystal SnS_2_ nanosheet	no	CVD	261	10^10^	[33]
Bi_2_S_3_ nanosheet	no	liquid-phase exfoliation	~10^−3^	~10^7^	[55]
WS_2_-CVD graphene	no	CVD	950	-	[56]
SnSe_2_ QD_S_-CVD graphene	no	liquid-phase exfoliation	7.5 × 10^3^	-	[22]
BP-CVD graphene	no	mechanical exfoliation	3.3 × 10^3^	-	[57]
PbS QD_S_-CVD graphene	yes	-	10^6^	-	[53]
BP NSs-CVD graphene	no	liquid-phase exfoliation	7.7 × 10^3^	-	[20]
CVD MoS_2_-CVD graphene	no	CVD	10^7^	-	[58]

Notes: The “Fabrication” means the preparation methods for light-sensitive materials except for the CVD-graphene.

## Data Availability

The data presented in this study are available on request from the corresponding author.

## Supplementary Materials

The following are available online at https://www.mdpi.com/article/10.3390/nano12030475/s1, Figure S1: Characterization of SnS_2_ nanosheets. (a) SEM images of SnS_2_ nanosheets distributed in the channel of the device. (b) Absorption spectra of SnS_2_ nanosheet solution. Inset: The photon energy dependence of (αhυ)^1/2^ to estimate the band gap; Figure S2: Detailed performance of graphene-PTAA-SnS_2_ photodetector at 405, 532 and 785 nm. (a), (b) and (c) Photocurrent of graphene-PTAA-SnS_2_ photodetector as a function of applied voltage at 405, 532 and 785 nm along with various radiant fluxes. (d), (e) and (f) Responsivity of graphene-PTAA-SnS_2_ photodetector as a function of applied voltage at incident wavelength of 405, 532 and 785 nm with varied light irradiance; Figure S3: Rectangular active area (the channel area) in different device units; Figure S4: Noise spectral density of 1/*f* noise vs frequency at V_DS_ = 0.5 V; Figure S5: Performance of the control sample of graphene-PTAA photodetector. (a) and (b) I–V curve of graphene-PTAA device as a function of V_DS_ (0.0–0.5 V) at 360 nm. (c) Time-dependent photoresponse of graphene-PTAA device on PET under periodic on/off illumination at wavelength of 360 nm; Figure S6: Mechanical stress of graphene-PTAA-SnS_2_ hybrid flexible device as a function of bending angles.
