# Supplementary material for: Highly-Responsive Broadband Photodetector Based on Graphene-PTAA-SnS2 Hybrid
Source: Nanomaterials (Basel). 2022 Jan 29;12(3):475. doi: 10.3390/nano12030475 (PMC8839128; doi:10.3390/nano12030475)
Supplement: Supplementary file 1 [file nanomaterials-12-00475-s001.zip › nanomaterials-1560525 supplementary materials final version.pdf]

Supplementary Materials

Highly-Responsive Broadband Photodetector Based on Graphene-PTAA-SnS_2 Hybrid

Guigang Zhou [1,2,†], Huancheng Zhao [3,†], Xiangyang Li [4], Zhenhua Sun [2], Honglei Wu [2], Ling Li [3,*], Hua An [1,2,*], Shuangchen Ruan [3] and Zhengchun Peng [1,2]

1 Center for Stretchable Electronics and NanoSensors, College of Physics and Optoelectronic Engineering, Shenzhen University, Shenzhen 518060, China; 2176285304@email.szu.edu.cn (G.Z.); zcpeng@szu.edu.cn (Z.P.)
2 Key Laboratory of Optoelectronic Devices and Systems of Ministry of Education and Guangdong Province, College of Physics and Optoelectronic Engineering, Shenzhen University, Shenzhen 518060, China; szh@szu.edu.cn (Z.S.); hlwu@szu.edu.cn (H.W.)
3 Shenzhen Key Laboratory of Laser Engineering, College of Physics and Optoelectronic Engineering, Shenzhen University, Shenzhen 518060, China; 1800281011@email.szu.edu.cn (H.Z.); scruan@szu.edu.cn (S.R.)
4 Key Laboratory of Advanced Optical Precision Manufacturing Technology of Guangdong Higher Education Institutes, College of Applied Technology, Shenzhen University, Shenzhen 518060, China; 2170285209@email.szu.edu.cn
* Correspondence: liling@szu.edu.cn (L.L.); huaan@szu.edu.cn (H.A.)
† These authors contributed equally to this work.

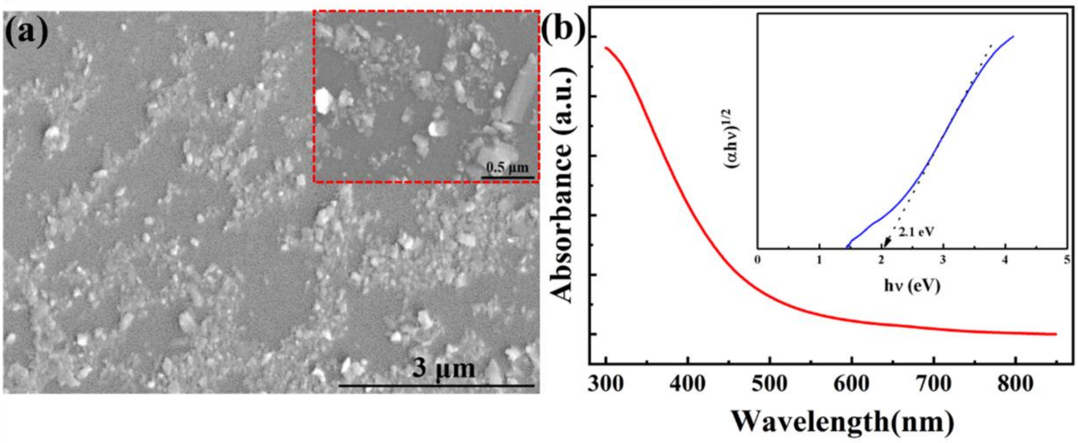

Figure S1. Characterization of SnS_2 nanosheets. (a) SEM images of SnS_2 nanosheets distributed in the channel of the device. (b) Absorption spectra of SnS_2 nanosheet solution. Inset: The photon energy dependence of $(\alpha h\upsilon)^{1/2}$ to estimate the band gap.

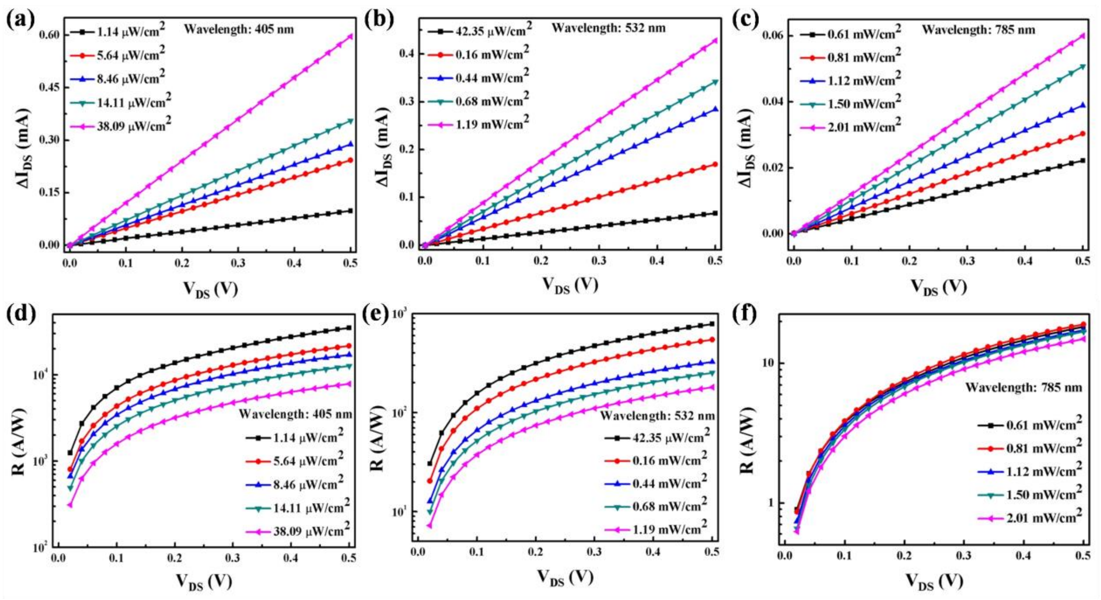

Figure S2. Detailed performance of graphene-PTAA-SnS_2 photodetector at 405, 532 and 785 nm. (a), (b) and (c) Photocurrent of graphene-PTAA-SnS_2 photodetector as a function of applied voltage at 405, 532 and 785 nm along with various radiant fluxes. (d), (e) and (f) Responsivity of graphene-PTAA-SnS_2 photodetector as a function of applied voltage at incident wavelength of 405, 532 and 785 nm with varied light irradiance.

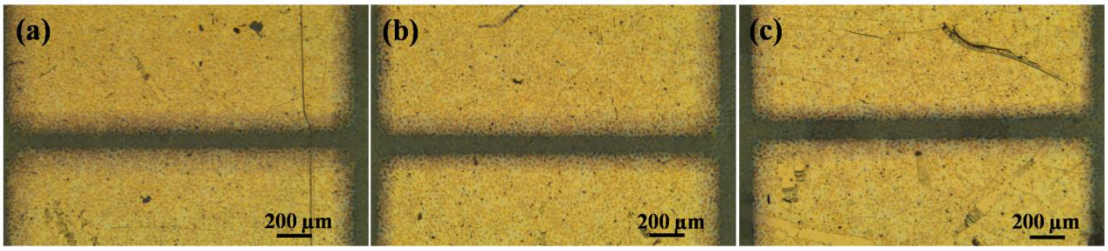

Figure S3. Rectangular active area (the channel area) in different device units.

Different sources of noise account for various contributions in the noise current. We have calculated the values of three noise sources (1/*f* noise, shot noise, thermal noise) derived from the literature [47-52]. The 1/*f* noise spectral density is given by $S_{If}(1/f) = \frac{|I(f)|^2}{F_S N}$, where I(*f*) represents the discrete Fourier transform of the measured dark current waveform I(t), and F_s and N are the sampling rate and number of data points, respectively. The resultant spectral density of 1/*f* noise dependent on the frequency is plotted in Fig S4. The 1/*f* noise spectral density is approximately 4.1×10^{-19} A^2 Hz^{-1} at a modulation frequency of 1 Hz. On the other hand, the shot noise spectral density is determined to be 2.65×10^{-20} A^2 Hz^{-1} using the formula of S_{Is} (shot) = $2qI_d$, where q and I_d are the electron charge and device dark current respectively. Moreover, the thermal noise spectral density is obtained with Nyquist's equation S_{It} (thermal)=$4k_0T/R$ (where k_0, T, R are the Boltzmann constant, temperature, and dark differential resistance of the device). The calculated S_{It} (thermal) is approximately 2.68×10^{-22} A^2 Hz^{-1}.

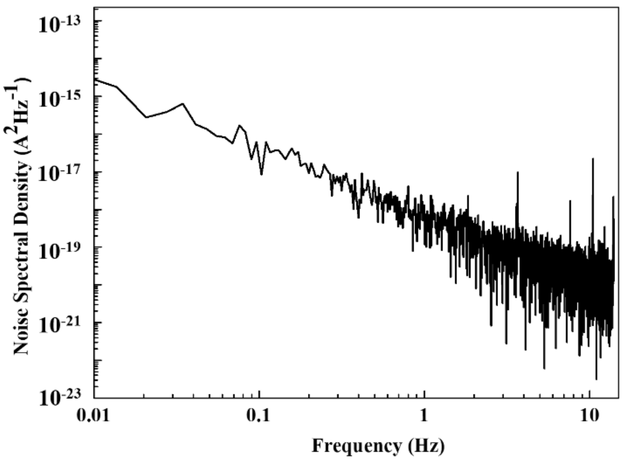

Figure S4. Noise spectral density of 1/*f* noise vs frequency at V_{DS} =0.5 V.

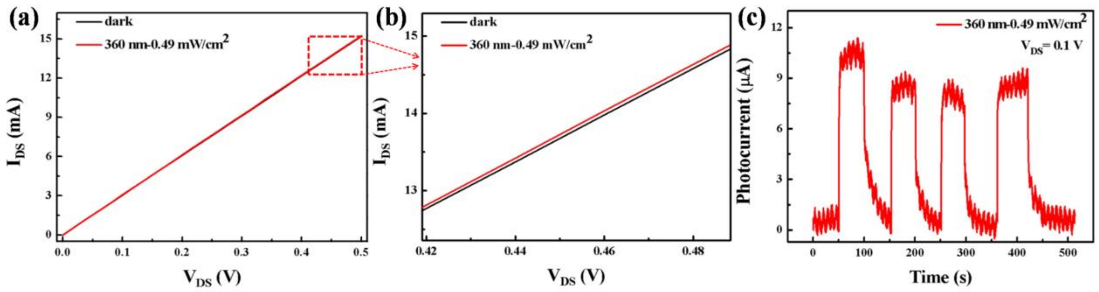

Figure S5. Performance of the control sample of graphene-PTAA photodetector. (a) and (b) I–V curve of graphene-PTAA device as a function of V_{DS} (0.0-0.5 V) at 360 nm. (c) Time-dependent photoresponse of graphene-PTAA device on PET under periodic on/off illumination at a wavelength of 360 nm.

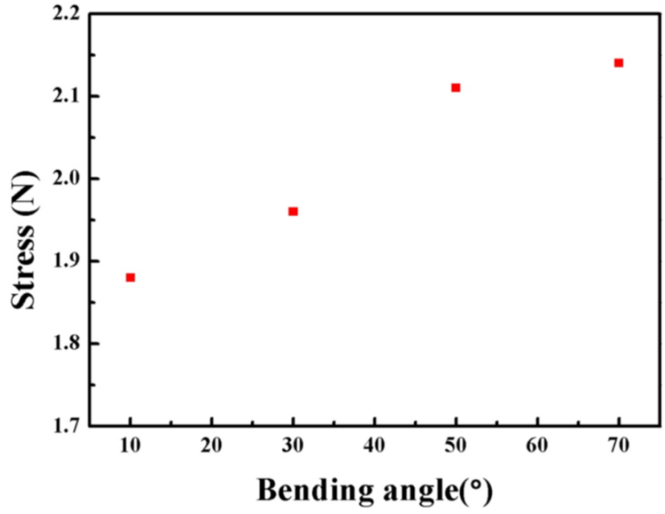

Figure S6. Mechanical stress of graphene-PTAA-SnS_2 hybrid flexible device as a function of bending angles.
